# A Novel Regulatory Player in the Innate Immune System: Long Non-Coding RNAs

**DOI:** 10.3390/ijms22179535

**Published:** 2021-09-02

**Authors:** Yuhuai Xie, Yuanyuan Wei

**Affiliations:** 1 Department of Immunology, School of Basic Medical Sciences, Fudan University, Shanghai 200032, China; yuhuai@fudan.edu.cn; 2 Shanghai Key Laboratory of Bioactive Small Molecules, State Key Laboratory of Medical Neurobiology, School of Basic Medical Sciences, Fudan University, Shanghai 200032, China

**Keywords:** long non-coding RNA, transcriptional regulation, inflammation, innate immunity, innate immune cells

## Abstract

Long non-coding RNAs (lncRNAs) represent crucial transcriptional and post-transcriptional gene regulators during antimicrobial responses in the host innate immune system. Studies have shown that lncRNAs are expressed in a highly tissue- and cell-specific- manner and are involved in the differentiation and function of innate immune cells, as well as inflammatory and antiviral processes, through versatile molecular mechanisms. These lncRNAs function via the interactions with DNA, RNA, or protein in either cis or trans pattern, relying on their specific sequences or their transcriptions and processing. The dysregulation of lncRNA function is associated with various human non-infectious diseases, such as inflammatory bowel disease, cardiovascular diseases, and diabetes mellitus. Here, we provide an overview of the regulation and mechanisms of lncRNA function in the development and differentiation of innate immune cells, and during the activation or repression of innate immune responses. These elucidations might be beneficial for the development of therapeutic strategies targeting inflammatory and innate immune-mediated diseases.

## 1. Introduction

The innate immune system is equipped with an arsenal of strategies to withstand infectious threats and maintain the normal activities and metabolism of the body. Activation of the innate immune system represents an immediate and initial response against pathogens and endows the body with the ability to repair and restore damaged tissue. Macrophages, dendritic cells, and granulocytes are important innate immune cells that participate in the immune response by sensing specific pathogen-associated molecular patterns (PAMPs) through their germline-encoded pattern recognition receptors (PRRs) [1]. The recognition of PAMPs by PRRs triggers an array of activation of intracellular signaling cascades, including adaptors, kinases, and transcription factors, leading to the expression of proinflammatory cytokines or antimicrobial genes [2]. However, activation of the inflammatory process can be a double-edged sword: although it is a crucial part of pathogen elimination, prolonged activation of these complex pathways might lead to tissue damage and diseases including cancer, cardiovascular diseases, and rheumatoid arthritis [2]. Thus, unsurprisingly, all of the aspects involved in inflammatory signaling pathways are tightly regulated at both the transcriptional and post-transcriptional levels [3].

Long non-coding RNAs (lncRNAs), which are defined as transcripts longer than 200 nucleotides and lacking protein-coding potential, represent the largest group of non-coding RNAs transcribed from the genome [4]. In the most recent LncRBase V.2 database release, 241,562 and 178,336 lncRNA transcripts were defined from the human and mouse genomes, respectively [5]. Advances in high-throughput technologies led to rapidly growing data accumulation in the identification of characteristics and functions of lncRNAs, which greatly expanded our understanding of the intricate and intriguing lncRNA biology. The gene-regulatory functions of lncRNAs not only depend on their specific transcript sequences but also on their own transcriptional process, which might account for the lower conservation of exon sequences but higher conservation of promoter regions in lncRNAs compared with that in protein-coding genes [6,7]. LncRNAs can regulate gene expression via multiple mechanisms at both the transcriptional and post-transcriptional levels [8]. Recent studies found that lncRNAs extensively participate in a series of biological and physiological processes, such as chromatin remodeling, cell cycle and proliferation, and metabolic homeostasis [9,10]. Moreover, lncRNA acts as a key regulator of innate immune responses and inflammation by activating various signal-dependent chromatin-modifying factors, transcription factors, and transcriptional coregulators [11]. However, the exact mechanisms of lncRNA bioactivities in innate immunity are still not completely clear.

Herein, we review the recent advances in illustration of the functions and mechanisms of lncRNAs in innate immune cell development and innate immune responses. In addition, we also discuss the important biological characteristics of lncRNAs, including their transcription, alternative splicing, cellular localization, and conservation. Despite the limited number of studies at present, we envision and believe that more intriguing lncRNAs and their biological functions will be exploited in the innate immune system in the near future.

## 2. Long Non-Coding RNAs

**Figure 1:**
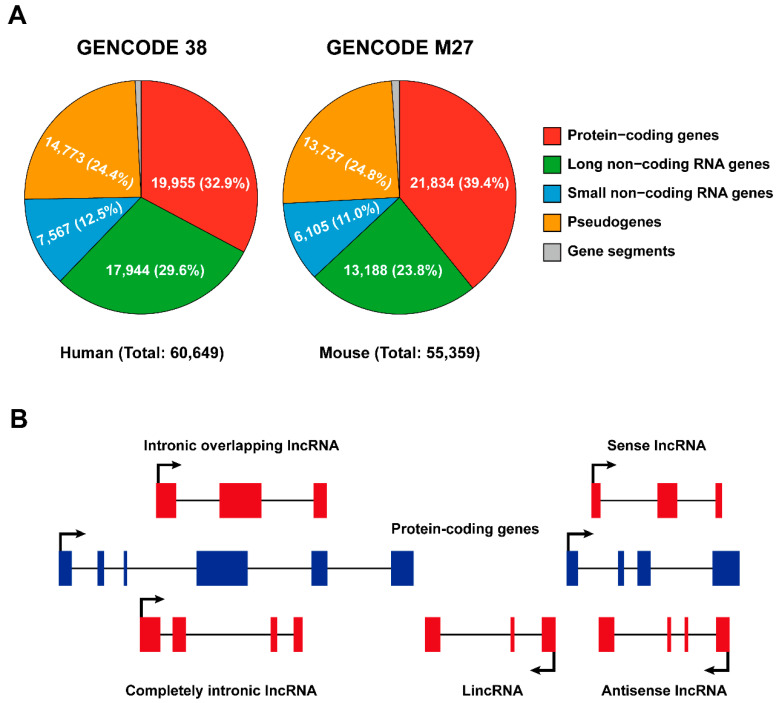
Classification of long non-coding RNAs. (**A**) Pie charts illustrating numbers and percentages of protein- and non-coding genes in the human and mouse genomes (released from GENCODE, 2021 update; https://www.gencodegenes.org (accessed on 5 May 2021)). (**B**) Classification of lncRNAs based on their localization with respect to genomic protein-coding genes. Sense/antisense lncRNAs are transcribed in the same/opposite direction as protein-coding genes and overlap at least one coding exon; completely intronic lncRNAs are transcribed from the intron of a protein-coding gene; intronic overlapping lncRNAs contain intronic sequences; lincRNAs are transcribed from regions between two protein-coding gene loci.

According to the Encyclopedia of DNA Elements (ENCODE) consortium (2012), the vast majority of the mammalian genome is transcribed [12,13]; nonetheless, only a small proportion (about 2%) of the genome is composed of genes that encode proteins, while the majority is transcribed as non-coding RNAs [7,14]. GENCODE represents the gene set of the ENCODE project and its most recent release (updated in 2021, https://www.gencodegenes.org (accessed on 5 May 2021)) indicates that there are 17,944 and 13,188 lncRNAs present in the human and mouse genomes, respectively (Figure 1A).

### 2.1. Classification of Long Non-Coding RNAs

In general, non-coding RNAs are categorized into lncRNAs and short non-coding RNAs according to a sequence length cutoff of 200 nucleotides. Based on their genomic location relative to enhancer elements or protein-coding loci, lncRNAs are further classified as enhancer RNAs (eRNAs), intronic lncRNAs, long intergenic non-coding RNAs (lincRNA), sense and antisense lncRNAs (Figure 1B). LncRNAs are often transcribed from either strand, within or outside non-protein-coding loci by RNA polymerase II (Pol II), and, like message RNAs (mRNAs), are capped, spliced, and polyadenylated [8,15]. LincRNAs refer to lncRNAs that are strictly intergenic and have no overlap with known protein-coding genes, while all other forms of lncRNAs overlapped with mRNA to varying degrees. eRNAs are particularly intriguing because they can be transcribed bidirectionally and capped from active enhancers but not spliced or polyadenylated [16]; however, some eRNA-liked lncRNAs transcribed from regulatory elements can be polyadenylated and unidirectional [17]. Antisense lncRNAs (Figure 1B) seem to be the primary lncRNA subtype, as it has been reported that over 70% of murine genomic loci are transcribed as antisense lncRNAs [18]. Although it was originally professed that lncRNAs are unstable, which is only true for a minority of lncRNAs, most can be stabilized through polyadenylation [19], and non-polyadenylated lncRNAs can also be stabilized depending on their secondary structures, such as hairpin motifs and triple-helical structures [20,21].

### 2.2. Transcription and Degradation of Long Non-Coding RNAs

Although lncRNAs and mRNAs have several similarities in their transcriptional process as mentioned above, large transcriptional differences have been identified between these two types of RNAs. Mammalian native elongation transcript sequencing (mNET-seq) data reveal that lincRNAs and mRNAs are transcribed by Pol II that differ in the phosphorylation state of the C-terminal domain (CTD). Usage of the Pol II isoform, which lacks phospho-CTD features associated with co-transcriptional splicing, and 3′ cleavage and polyadenylation, allows for lincRNAs with lower levels of co-transcriptional splicing and inefficient polyadenylation compared with pre-mRNAs [22]. Moreover, different from pre-mRNAs, lincRNAs are mostly restricted to chromatin, partially due to interaction with the U1 snRNP. These lincRNA are degraded by the nuclear RNA exosome, resulting in few lincRNAs being detected in cytoplasm [23]. However, the reasons that chromatin-associated lincRNAs rapidly degrade after transcription remain elusive.

In addition to the rapid post-transcriptional degradation, and low expression of lncRNAs [24], as well as their tissue- and cell-type specificity, they are directly associated with DNA methylation and histone modification. Mammalian promoters can be categorized into two types according to their CpG dinucleotide content: high CpG (HCG) and low CpG (LCG) [25]. The HCG class of promoters is hypomethylated, while the LCG class of promoters is hypermethylated, and the latter generally repress gene transcription. In the mammalian genome, a large proportion of lncRNAs are transcribed from the LCG class of promoters, of which only 6.5% are marked by histone H3K4me3, associated with higher transcription activity, resulting in low expression levels for lncRNAs. Moreover, the abundance of CpG dinucleotide usually has a negative correlation with the potential for chromosome condensation, indicating that lncRNA promoters may be highly condensed and unsuitable for transcription [26]. Moreover, enrichment of transcription factor binding sites [27] and specific DNA sequence (e.g., CCG and CGG repeats) in lncRNA loci [28] are significantly correlated with lncRNA expression. In addition, miRNA-dependent regulation of promoter methylation leads to the complexity of dynamic expression of lncRNAs during diseases [29].

Considering the short history of lncRNA studies, more detailed transcriptional features of lncRNAs should be investigated further—for example, researchers must establish whether lncRNA transcription depends on other regulatory non-coding RNAs.

### 2.3. Alternative Splicing of Long Non-Coding RNAs

Similar to protein-coding mRNAs, the vast majority of lncRNAs undergo extensive alternative splicing, which greatly increases their potential number of isoforms [30]. Overall, lncRNAs are spliced less efficiently than mRNAs, and the splicing frequency of specific introns in lncRNAs is usually more variable compared with that of mRNA introns [31], leading to substantially more alternative splicing isoforms of lncRNAs. Moreover, different from mRNAs, there is no need to maintain open reading frames (ORFs), which may allow the spliceosome to explore the full spectrum of exon combination, resulting in a high diversity of lncRNA isoforms [30]. In addition, recent reports have also proved that lncRNAs can act as the precursors of miRNAs. The exon alternative splicing of lncRNAs can produce important conserved miRNAs [32]. The alternative splicing of either protein-coding or non-coding RNAs is regulated by a comprehensive list of cis-regulatory elements and trans-regulatory factors. These cis-regulatory elements contain intron-splicing enhancers (ISEs) and silencers (ISSs), and exon-splicing enhancers (ESEs) and silencers (ESSs), while trans-acting factors include Ser/Arg-rich (SR) proteins and heterogeneous nuclear ribonucleoproteins (hnRNPs), which function via binding to cis-acting sequences [33,34]. SR proteins are generally considered positive splicing regulators and promote exon inclusion by recruiting U1 snRNP and U2 auxiliary factors to exons; in contrast, hnRNPs, including hnRNP A/B and PTB, are viewed as negative for cis-acting elements [34]. These extensive alternative splicings, as a complex and overlooked aspect of lncRNAs, might further diversify the potential biological function of lncRNAs, along with newly identified lncRNA partners or localization patterns, due to the specific exon involvement; however, this is largely unknown. 

### 2.4. Conservation and Secondary Structure of Long Non-Coding RNAs

At the sequence level, researchers found that lncRNAs are, overall, much less conserved compared with protein-coding genes [35,36]. The poor sequence conservation and low abundance of lncRNAs initially led some researchers to suggest that most lncRNAs may represent transcriptional “noise” and have little biological significance [37]. Nevertheless, some studies showed that >85% of human GENCODE lncRNAs can be dated back to the divergence of placental mammals according to the conserved splice sites [38]. Despite the fast turnover of exon/intron structures that was observed, the exons show higher conservation than introns in human lncRNAs [7].

Secondary structure is one of the crucial factors determining the function of lncRNAs, as supported by several classical functionally characterized lncRNAs in the previous analyses. For instance, the functions of *MEG3* for the activation of p53 signaling and suppression of tumor cell growth can be attributed to a secondary folding motif, identified using an RNA secondary structure prediction program, Mfold [39]. It has also been shown that *MALAT1* requires an intact U-rich stem loop duplex-triplex and A-rich tract to maintain its RNA stabilization activity [20]. Overall, wet experiments suggest that lncRNAs exhibit a higher degree of secondary folding than that predicted by algorithms, despite the fact that lncRNAs seem to be less structured than mRNAs [40]. These specific secondary structures may play a decisive role in lncRNA bioactivities.

### 2.5. Subcellular Localization of Long Non-Coding RNAs

The subcellular localization of lncRNAs is very important, as it provides critical information for the understanding and prediction of, as well as investments in, non-coding action patterns, including associated molecules, post- or co-transcriptional regulatory modifications, and external stimuli directly affecting lncRNA functions. Unlike mRNAs, which are translated into proteins in the cytoplasm regardless of their functions, lncRNAs must localize where they play specific roles. In accordance with this, studies indicate that nuclear lncRNAs are more abundant than cytoplasmic ones probably due to their main functions in nuclear architecture organization [41,42,43], although the number of cytoplasmic lncRNAs is gradually expanding. However, nuclear lncRNAs seem to be more instable due to their low expression and the involvement of unstable transcripts, such as upstream promoter transcripts. This might reflect their specific function in gene expression, as well as transcriptional or post-transcriptional regulation of gene expression [19]. Several RNA sequence motifs have been identified as responsible for nuclear localization, such as C-rich motifs outside Alu-like elements in the sequence of some lncRNAs [44]. The repeat E motif was also found to be involved in the localization of *lncRNA Xist* [45]. In addition to their own characteristics, including genomic and subcellular localization, GC percentage and splicing, the instability of nuclear lncRNAs can also be regulated by the poly (A) binding protein PABPN1, which promotes poly (A)-polymerase (PAPα/β)-dependent hyperadenylation and subsequent decay [46]. 

Besides nuclei, studies also have begun to interrogate the localization of lncRNAs to specific macromolecular structures or organelles. This localization of RNA has been largely studied using fractionation-based methods combined with RNA-seq and fluorescence in situ hybridization (FISH) [47,48]. APEX is an engineered peroxidase and can catalyze biotin-phenol and hydrogen peroxide to form biotin-phenoxyl radicals. These radicals can then diffuse outward and covalently biotinylate the adjacent endogenous proteins, but not the distal proteins, because of their extremely short half-life. Therefore, a method in living cells that combines an engineered APEX that targets the cellular compartment of interest [49,50] with RNA immunoprecipitation (RIP) has allowed for the identification and quantification of RNAs localized in varieties of subcellular compartments, including the nucleus, cytosol, mitochondrial matrix, and endoplasmic reticulum (ER) [41]. Another RNA aptamers (consisting of Tat peptide and trans-activation response (TAR) element) and fluorogenic proteins system also provided a pipeline to visualize RNA localization in living cells [51]. In this system, a bifunctional peptide (termed “tDeg”), containing a Tat peptide and degron sequence, is fused to the fluorogenic protein. The RNA aptamers (termed “Pepper”) inserted into the RNA of interest can bind to the Tat peptide, preventing degron from recruiting proteasome and stabilizing the fluorogenic protein. Thus, the RNA of interest can be detected by the fluorescence signal. Based on these established methods, a small portion of organelle-related lncRNAs have been identified and further functionally characterized. Moreover, cellular localization of lncRNAs can be predicted using a publicly available web server iLoc-LncRNA (http://lin-group.cn/server/iLoc-LncRNA (accessed on 15 Dec 2018)) [52], which can be considered as the first step for researchers attempting to predict the localization of their candidates according to their sequences.

## 3. Long Non-Coding RNAs Function as Transcriptional Regulators

**Figure 2:**
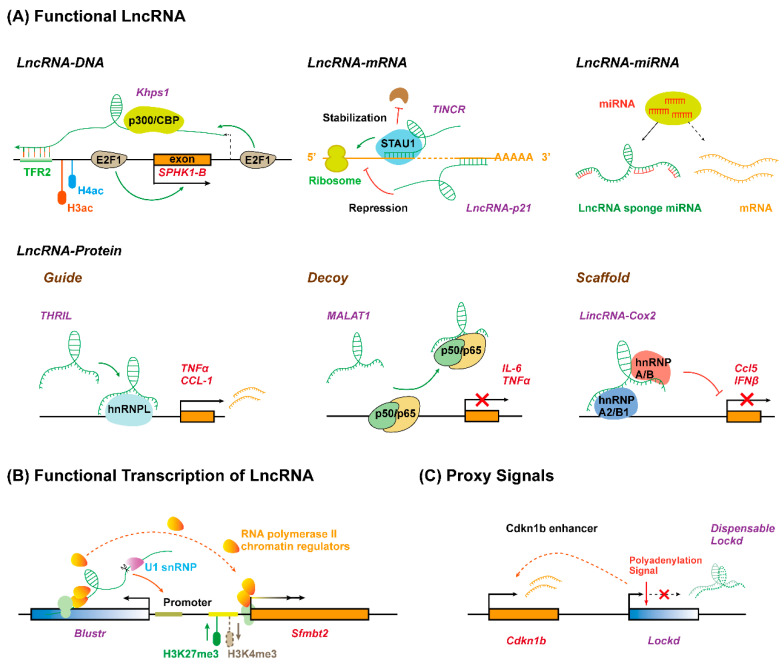
Long non-coding RNAs function as versatile transcriptional regulators in three different manners. (**A**) LncRNA molecule is functional due to its specific sequence interacting with DNA, RNA, or protein. LncRNA (*Khps1*) tethers to DNA via formatting triplex with specific sequence and guides chromatin regulators to target gene (*SPHK1-B*) promoter. LncRNA interacts with target mRNA by base-pairing to repress (*lncRNA-p21*) or enhance (*TINCR*) translation. LncRNA sponges miRNA to inhibit degradation and translation repression of mRNA. LncRNA interacts with specific proteins to regulate target gene expression by acting as a guide (*THRIL*), decoy (*MALAT1*), or scaffold (*lincRNA-Cox2*). (**B**) The act of lncRNA transcription is functional. The transcription and promoter-proximal splicing process of lncRNA (*Blustr*) alter the chromatin state and ensure RNA polymerase elongation at the promoter of target gene (*Sfmbt2*). (**C**) LncRNA acts as proxy signal for cis-regulatory elements. The 5′region or promoter of lncRNA (*Lockd*) contains an enhancer for the neighboring genes (*Cdkn1b*). TFR, triplex-forming regions.

Although the biological functions of lncRNAs are just starting to be studied and understood, it has been known that lncRNAs play critical roles in almost every biological process mainly through three different patterns: the lncRNA molecule itself is functional depending on its specific sequence; the process of lncRNA transcription, rather than the lncRNA molecule itself, has a function; lncRNA functions as proxy signals for active cis-regulatory elements (Figure 2) [53]. During these processes, it has been proposed that lncRNAs can exert regulatory roles, either in cis or in trans, by serving as molecular signals, decoys, guides, and scaffolds, through interacting with DNA, RNA, or proteins [54]. However, given their exquisite cell-type-specific expression pattern and poor sequence conservation, it remains to be understood whether lncRNAs have new action patterns besides these three mechanisms 

### 3.1. Functional Long Non-Coding RNA Molecules

The most common method through which to study the function of lncRNAs is to characterize the direct biological activity of the lncRNA molecule itself. Most lncRNAs discovered to date modulate transcription by binding to the target DNA in cis (for neighboring genes) or trans (for distal genes) through the recognition of specific chromatin features (Figure 2A). They can interact with single-stranded or double-stranded DNA by forming RNA–DNA hybrid duplex or RNA–DNA triplex structures through Watson–Crick or Hoogsteen hydrogen bonding [54,55]. For example, a lncRNA, *Khps1*, can anchor to proto-oncogene *SPHK1* promoter by forming a DNA–RNA triplex with a homopurine stretch upstream of the transcription start site. Then, *Khps1* recruits the histone acetyltransferase p300/CBP to the *SPHK1* promoter, changing the chromatin structure and facilitating *SPHK1* transcription in cis by ensuring the binding of transcription factor E2F1 [56]. CTCF is one highly conserved zinc finger protein and can coordinate chromatin structures to regulate gene expression [57]. LncRNA *CCAT1-L* localizes at the transcription site, spatially close to the *MYC* oncogene, and maintains the chromatin looping between the *MYC* promoter and its enhancers in coordination with CTCF, thus, enhancing *MYC* transcription in cis [58]. *HOTAIR* transcribed from the *HOXC* locus has been demonstrated to interact with methyltransferase Polycomb repressor complex 2 (PRC2) subunits (including Ezh2 and Suz12) [59] and functions as a bridge by recruiting PRC2 to the gene loci to promote target gene silencing through a complex array of post-translational modifications of histones in trans [60]. These studies also indicated that lncRNAs can regulate gene transcription by acting as a bridge between chromatin-modifying proteins and chromatin modification elements. The heterogeneous nuclear ribonucleoproteins (hnRNPs) are predominant nuclear RNA-binding proteins that form complexes with RNA polymerase II transcripts, which function in the transcription, processing, and translation of mRNA [61]. A growing body of research has indicated that lncRNAs, such as *lincRNA-p21* (hnRNP-K) [62], *lncRNA-ITPF* (hnRNP-L) [63], *LincRNA-Cox2* (hnRNP-A/B) [11], and *lncRNA ST3GAL6-AS1* (hnRNPA2B1) [64] can recruit transcriptional machinery to the promoters of target genes via association with hnRNPs [62]. 

In addition to regulating gene transcription, lncRNAs containing miRNA response elements (MREs) can regulate protein-coding mRNAs harboring the same MREs by acting as competing endogenous RNAs (ceRNAs) or natural microRNA sponges at the post-transcriptional level [65,66]. The competitive binding of these ceRNAs to the seed region of the shared miRNAs results in derepression of other RNA transcripts that contain the same MREs [66]. Extensive transcriptome data have revealed that a large repositories of lncRNA/miRNA pairs, such as *MALAT1*-*miR-181c-5p/miR-125b/miR-146a/miR-199b* [67,68,69,70], *lincRNA-Cox2-let-7a/miR-150-5p* [71,72], *RP11-86H7.1-miR-9-5p* [73], *SNHG5-miR-132* [74], play an important role in inflammation and innate immunity via the ceRNA regulatory network. 

Future studies on lncRNA structures and motifs will contribute to a better understanding of the mechanisms by which lncRNAs interact with specific DNA, RNA, and proteins, as well as how lncRNAs localize at specific sites.

### 3.2. Functional Roles of the Act of Long Non-Coding RNA Transcription 

In some contexts, the biogenesis process for a lncRNA but not the lncRNA itself can impact the expression of nearby genes (in a cis pattern), because the process of transcription or splicing of lncRNAs may recruit specific protein factors (e.g., transcription factors, repressor proteins, and polymerase) or remodel nucleosomes, which regulates gene transcription [75,76]. Genetic manipulation in mouse cell lines found that five genomic loci that produce lncRNAs influence the expression of the neighboring gene in cis and, intriguingly, all of these effects do not require the specific lncRNA transcripts themselves, while they do involve the general processes associated with their production, including the enhancer-like function of their promoters, the transcription process, and/or the splicing of these transcripts (Figure 2B) [77]. Deletion of the promoter of lncRNA *Bendr* (*linc1536*) decreased the expression of the nearby protein-coding gene *Bend4* by 57%; however, the effects require neither a mature nor a significant amount of the *Bendr* transcript [77]. In addition, an increase in the length of lncRNA *Blustr* (*linc1319*)-transcribed region by engineered pAS insertions promoted the activation of *Sfmbt2*, located 5 kB upstream, independent of any specific sequence elements in mature *Blustr*. Moreover, the first 5′ splicing site of *Blustr* plays a vital role in activating *Sfmbt2* transcription, probably because the splicing event leads to the recruitment of transcriptional machinery acting on the nearby *Sfmbt2* promoter [77]. 

Although it is becoming clearer that the production process of lncRNAs influences neighboring gene transcription, how transcription and splicing across lncRNA loci recruit the vital regulatory elements or change the dynamic of chromatin to coordinate gene expression in cis remains to be illustrated.

### 3.3. Long Non-Coding RNAs Act as Proxy Signals for Cis-Regulatory Elements

In addition to the production process of lncRNA transcripts, certain conserved lncRNA promoters may regulate transcription of the adjacent genes as cis-acting enhancer elements (Figure 2C) [78]. In this case, lncRNA transcripts are non-functional byproducts marking the regulatory activity of their promoters. For example, the deletion rather than truncation of lncRNA *Lockd* impairs the transcription of its upstream coding gene *Cdkn1b*, due to the enhancer-like DNA elements within the *Lockd* promoter [79]. Therefore, the transcription of lncRNAs might act as proxy signals of the activity of crucial DNA regulatory elements. 

The prevalence of the three functional mechanisms mentioned above suggests two patterns for the evolutionary selection of lncRNAs: one is restricted to the RNA sequence while in another, functional cis-regulatory elements but not the sequence of lncRNAs are implicated [24,35]. This raises the possibility that, despite the limited sequence conservation, some lncRNAs have conserved functions across species. 

## 4. Long Non-Coding RNAs Function in Innate Immunity

In biological immune responses, the innate immune system serves as the initial defense against foreign and harmful substances. Both professional innate immune cells, including macrophages, mast cells, natural killer (NK) cells, neutrophils, eosinophils, basophils, and dendritic cells (DCs), and nonprofessional innate immune cells, such as endothelial cells, and fibroblasts [2], undergo immediate rapid changes in gene expression and regulation programs to respond to pathogenic invasion, tissue damage, stress, and metabolic dysregulation [80]. Although pathogens can evolve rapidly, the innate immune system can always detect the invading pathogens and common biologic consequences of infection relying on a limited repertoire of receptors. Innate immune cells have evolved to target conserved microbial components that are shared by most pathogens to compensate for the limited number of receptors. To enlarge cellular defenses, the innate immune system also contains many humoral components, including well-characterized components, such as C-reactive protein, complement proteins, and lipopolysaccharide (LPS) binding protein, and less-well-studied antimicrobial peptide components. 

Given their role in mediating gene transcription and translation, a growing body of discoveries has revealed that lncRNAs are excellent candidates for the regulation of the mammalian innate immune processes, including the clearance of bacterial and viral infection, host inflammatory responses, and development of manifold innate immune-mediated diseases, in both positive and negative patterns [81]. These findings have served as an impetus for a further and thorough exploration of how lncRNAs regulate the innate response as well as the sophisticated immune cell development. We will focus on lncRNAs that are recently identified and widely studied and review their functions and underlying mechanisms according to their dependent signaling molecules and patterns of action.

### 4.1. Long Non-Coding RNAs in the Development of Innate Immune Cells

In recent years, lncRNAs have merged as regulators of somatic cell differentiation in tissues ranging from epidermal to adipose tissues [82,83], as well as osteogenic differentiation of mesenchymal stem cells [84], while their biology and function in the development, differentiation, and maturation of professional innate immune cells are only beginning to be explored. Innate immune cells are generated from hematopoietic stem cells (HSCs) and consist of myeloid cells derived from mononuclear phagocytes (e.g., macrophages, differentiated from blood monocytes) and polymorphonuclear phagocytes (e.g., granulocytes), and lymphoid lineage cell-derived NK cells [85]. While the adaptive immune system mainly includes T and B lymphocytes, innate immune cells are vital in the immune system because they support the functions of the adaptive immune system, depending on the production of cytokines and the antigen-presenting function [86]. 

#### 4.1.1. Macrophages

Macrophages belong to the mononuclear phagocyte system, defined by their origin from bone-marrow-derived cells, and their phagocytosis, cytokine secretion, and antigen presentation abilities. Cells of the mononuclear phagocyte system have a great capacity to specialize, particularly during inflammatory response, where monocytes are recruited into the tissues and differentiate into macrophages. Macrophages are vital cells for innate immune sensing, accomplished by Toll-like receptors (TLRs) on their surface, and are considered as the first line of the host innate immune system [87]. It has been found that in the process of monocyte/macrophage differentiation of THP-1 cells and CD34^+^ HSPCs, *lnc-MC* (Table 1) promotes the differentiation process by sequestering *miR-199a-5p* and releasing the expression of activin A receptor type 1B (*ACVR1B*), an important regulator of monocyte/macrophage differentiation [88]. Overexpression of *PBOV1* in THP-1 cells results in their differentiation into macrophages, and an RNA IP assay showed that lncRNA *NTT* could upregulate *PBOV1* expression by interacting with hnRNP-U binding to the promoter of *PBOV1* [89].

In response to various pathogen- and self-local-environment-derived stimuli, macrophages exhibit a strong phenotypic and functional plasticity and complexity [90], where M1 (classically activated macrophages) and M2 (alternatively activated macrophages) represent two extreme macrophage subtypes in vitro [91]. Functionally, M1 macrophages that are activated by bacterial LPS and interferon-γ (IFN-γ) produce abundant amounts of proinflammatory cytokines (such as TNF-α, NO, IL-1, IL-12, and IL-23) or reactive oxygen species (ROS) to kill pathogens and promote Th1 immune response. TLR-triggered NF-κB is one of the well-studied pathways that participates in the polarization of macrophages to M1 phenotype [92]. Transcriptome analysis has shown that the expression levels of numerous lncRNAs are altered in macrophages upon stimulation of TLR ligands including LPS. Of note, several LPS-regulated lncRNAs, such as *lncRNA-NfκB2* and *lncRNA-Rel*, are located near to proinflammatory protein-coding genes, indicating their potential role in regulating M1 macrophage polarization [93]. 

However, in the presence of granulocyte macrophage colony stimulating factor (GM-CSF), IL-4, IL-10, IL-13, or immune complexes (ICs) together with either TLR or IL-1R ligands, macrophages tend to polarize into M2 subtypes, which subsequently leads to an anti-inflammatory Th2 response, thus, enhancing tissue repair and remodeling [94]. Microarray analysis found 264 upregulated and 289 downregulated lncRNAs in IL-4 induced M2 macrophages. Additionally, *PTPRE-AS1*, one of these potently enhanced lncRNAs, acts as a repressor of M2 activation by activating *PTPRE* through the recruitment of WDR5 to the *PTPRE* promoter [95]. As one of the most highly induced lncRNAs in macrophages by TLR activation, *lincRNA-Cox2,* located downstream of protein-coding *Cox2*, is required for the NF-κB-mediated transcription of proinflammatory genes [11], whereas it inhibits M2 polarization [96]. LncRNA growth-arrest-specific 5 (*GAS5*) shifts macrophages toward the M1 subtype from the M2 subtype by acting as a *miR-455-5p* ceRNA regulator that promotes *SOCS3* expression during childhood pneumonia [97]. Similar to *GAS5*, *MIR-155HG* induces M1 macrophage polarization, whereas it impedes M2 polarization, albeit through an unknown underlying mechanism [98]. In contrast, lncRNA *Mirt2* acts as a negative regulator of LPS-activated inflammatory response by suppressing NF-κB and MAPK pathways in macrophages [99], and *lncRNA-MM2P* promotes cytokine-stimulated M2 polarization by enhancing signal transducer and activator of transcription 6 (STAT6) phosphorylation [100]. Despite the fact that the lncRNA has been shown to be a key regulator of macrophage polarization, there is still substantial room for research, especially in vivo.

**Table 1:** Long non-coding RNAs in the development and polarization of innate immune cells.

LncRNA	Target Genes	Functional Consequences	Mechanism	Reference
Lnc-MC	miR-199a-5p	Promotes macrophage differentiation	Releases ACVR1B	[88]
NTT	PBOV1	Promotes macrophage differentiation	Recruits hnRNP-U to PBOV1 promoter	[89]
LincRNA-Cox2	NF-κB-mediated cytokines	Inhibits M2 polarization	-	[96]
GAS5	miR-455-5p	Promotes M1 polarization from M2	Release SOCS3	[97]
MIR-155HG	Proinflammatory cytokines	Induces M1 polarization	-	[98]
Mirt2	TRAF6	Promotes M2 polarization	Suppresses NF-κB and MAPK pathway	[99]
LncRNA-MM2P	STAT6	Promotes M2 polarization	Increases phosphorylation of STAT6	[100]
PTPRE-AS1	PTPRE	Inhibits M2 activation	Recruits WDR5 to PTPRE promoter	[95]
Lnc-DC	STAT3	Promotes DCs differentiation	Prevents Y705 dephosphorylation of STAT3 by SHP1	[101]
MALAT1	miR-155	Induces tolerogenic DCs	Releases DC-SIGH and IL-10	[102]
HOTAIRM1	HOXA cluster, CD11b and CD18	Promotes granulocyte differentiation and maturation	-	[103]
Lnc-CD56	CD56	Promotes CD56 NK cell development	-	[104]
GAS5	miR-544	Enhances CD107a+ NK cells and its cytotoxicity	Upregulates RUNX3 as a sponge	[105]
Linc-EPHA6-1	has-miR-4885-5p	Promotes cytotoxicity of NK cells	Upregulates NKp46 expression as a sponge	[106]

#### 4.1.2. Dendritic Cells

In the same way as macrophages, DCs originate from the mononuclear phagocyte system, and bridge the innate and adaptive arms of the immune system by acting as the primary antigen-presenting cells (APCs) for T lymphocytes [107]. DCs are classified into two subtypes: conventional DCs (cDCs) that function as APCs, and plasmacytoid DCs (pDCs) that produce copious levels of the type I IFN against viral and bacterial infections [108]. RNA-seq analysis at different stages of monocyte differentiation into DCs identified a cohort of regulated lncRNAs involved in DC maturation. A lncRNA that is exclusively expressed in human DCs, named *lnc-DC*, is vital for DC differentiation from both human monocytes and mouse bone marrow cells through controlling the expression of DC markers CD40, CD80, CD86, and HLA-DR by binding to the transcription factor STAT3 [101]. In addition, *lnc-DC* deficient DCs failed to take up antigens and induce allogeneic CD4+ T cell proliferation and cytokine production [101]. LncRNA *MALAT1* can induce tolerogenic DCs via the prevention of *miRNA-155* targeting of *DC-SIGH* and *IL-10* as a sponge [102].

#### 4.1.3. Granulocytes

The *HOXA* gene cluster is a homeotic gene that encodes a family of transcription factors that participate in the establishment and maintenance of cellular identity in embryogenesis [109]. The intergenic non-coding transcript HOX antisense intergenic RNA myeloid 1 (*HOTAIRM1*) located between the *HOXA1* and *HOXA2* genes has been demonstrated to be associated with granulocytic differentiation and maturation [103]. *HOTAIRM1* expression is specific to the myeloid lineage and is upregulated during the retinoic acid (RA)-promoted granulocytic differentiation of NB4 promyelocytic leukemia and human normal hematopoietic cells. In addition, the shRNA-mediated knockdown of *HOTAIRM1* attenuated the transcriptional induction of its neighboring genes at the 3′ end of the *HOXA* cluster and impeded the transcription of genes encoding β2 integrins CD11b and CD18. Moreover, the association of *HOXA* genes with the transcriptional regulation of normal hematopoiesis [110] and acute myeloid leukemia [111] indicates that *HOTAIRM1* may additionally play functional roles in myelopoiesis via regulating *HOXA* expression in cis.

#### 4.1.4. Natural Killer Cells

Different from macrophages, DCs, and granulocytes of myeloid origin, NK cells featuring CD3-negative and CD56-positive surface markers are innate lymphoid cells with cytotoxic effects [112] and produce various cytokines in response to bacterial, viral, and parasitic infections [80]. Recent studies demonstrated that lncRNAs play important roles in the development and function of NK cells. An example of a prototypical lncRNA with cis regulatory function in NK cells is *lnc-CD56* [104], which is highly expressed in human CD56^bright^ NK cells, and has a superior ability to produce proinflammatory cytokines in comparison with more cytotoxic CD56^dim^ NK cells [113]. Knockdown of *lnc-CD56* reduced CD56 expression and decreased mature CD56^bright^ NK cell content, demonstrating the requirement of *lnc-CD56* for CD56 maintenance during NK cell development [104]. Another lncRNA *GAS5* was found to be downregulated in the NK cells of liver cancer patients, which causes a reduction in IFN-γ production, a decrease in the percentage of CD107a^+^ NK cells and the impaired cytotoxicity of NK cells, attributed to *RUNX3* upregulation by sponging *miR-544* [105]. Recent research has also showed that exosomal *linc-EPHA6-1*, induced by IFN-β, can promote NKp46 expression and cytotoxicity of NK cells through its interaction with *has-miR-4885-5p* [106]. 

Together, these studies demonstrate the importance of lncRNAs in controlling the development of innate immune cells. Nevertheless, more research should be carried out to decipher the mechanisms underlying the roles of lncRNA involved in innate immune cell differentiation and polarization, as well as their functions.

### 4.2. Long Non-Coding RNAs Function in Host Inflammatory Response Triggered by the Innate Immune System

#### 4.2.1. Inflammatory Signaling Triggered by PAMPs and DAMPs

**Figure 3:**
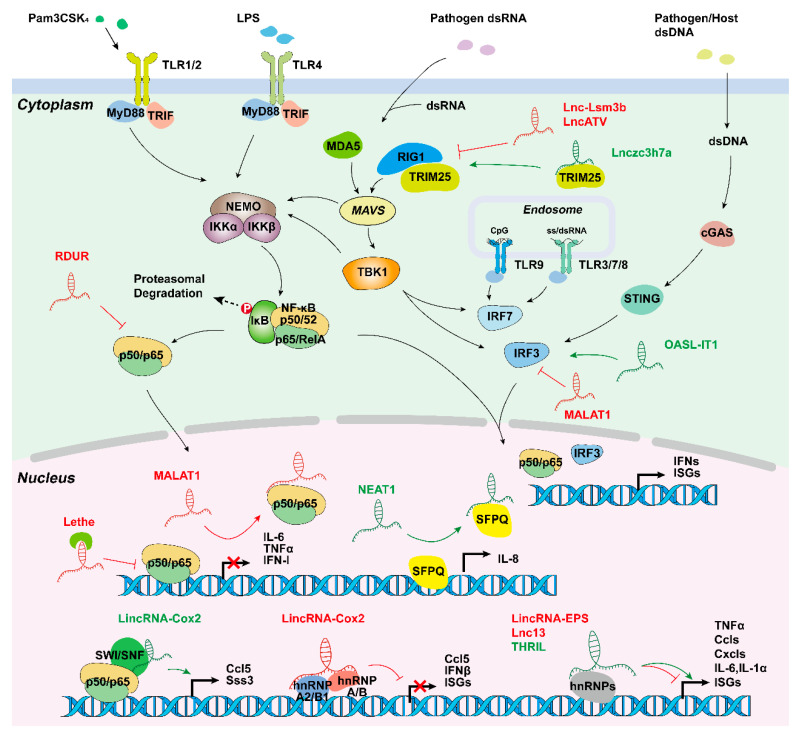
Long non-coding RNAs involved in innate immune responses. *LincRNA-Cox2* activates or represses inflammatory genes through SWI/SNF-NF-κB complex or interactions with hnRNP-A/B and hnRNP-A2/B1; *Lethe* inhibits NF-κB locating target gene promoters; *MALAT1* creates RNA-protein complex; *NEAT1* initiates transcription by transferring SFPQ; and several other lncRNAs are involved in inflammation and virus-mediated innate immune response. hnRNP, heterogeneous nuclear ribonucleoprotein; SWI/SNF, switch/sucrose.

The innate immune response is initiated by the binding of microbial structures, termed pathogen-associated molecular patterns (PAMPs), to PRRs on the surface of innate immune cells [2]. The second defensive approach used by the innate immune system is the to detection of an immunological danger signal in the form of damage-associated molecular pattern molecules (DAMPs), such as high-mobility group box 1 protein, heat shock proteins, and uric acid, that are released from infected or damaged host cells [114]. Based on their protein domain-structure, PRR families are classified into four classes: Toll-like receptors (TLRs), C-type lectin receptors (CLRs), Retinoic acid-inducible gene (RIG)-I-like receptors (RLRs), and NOD-like receptors (NLRs) [2]. Different PRR family members can recognize diverse pathogen structures ranging from bacterial and viral nucleic acids, such as unmethylated CpG DNA (TLR9), dsRNA (e.g., TLR3, MDA5, Rig-I), or cytosolic DNA (cGAS), to bacterial cell wall components, such as bacterial lipoproteins (TLR2), lipopolysaccharides (TLR4), and peptidoglycans (NLRs) [2,115]. When PRRs sense the presence of PAMPs or DAMPs, the transcription of genes encoding proinflammatory cytokines (e.g., TNF-α, IL-1, IL-6), chemokines (e.g., CCL2, CXCL8), interferons, antimicrobial proteins, or proteins involved in the modulation of PRR signaling are upregulated by the activation of master-transcription factors during the immune response, such as NF-κB, MAPK, and STAT protein families (Figure 3) [116].

The NF-κB family contains seven distinct members, including NF-κB1 (p105 and p50), NF-κB2 (p100 and p52), RelA (p65), RelB and c-Rel, which can form a variety of dimers by interacting with each other [117]. TLR4 signaling leads to IKK complex activation and the subsequent phosphorylation and degradation of the inhibitor of NF-κB (IκB), which frees NF-κB that enters the nucleus and binds to a DNA motif, named the response elements (RE), initiating inflammatory gene transcription. Additionally, TLRs recruit IRAK and TRAF6, causing the activation of TGF-activated kinase 1 (TAK1) and MAPK family members (including p38, JNK, and ERK1/2), which mainly contributes to the expression of inflammatory mediators [118]. TAK1 can also activate the IKK complex leading to the release of NF-κB [119]. STAT3, a member of the STAT family, is another important transcriptional factor involved in immune responses, inflammation, and tumorigenesis. STAT3 activation requires the phosphorylation of Tyr705, which can be mediated by Janus kinases (JAKs, especially JAK2) [120] or activated by ROS accumulation [121], and then promotes the activation of inflammatory pathways, such as the NF-κB and IL6-GP130-JAK pathways [122].

Thus, NF-κB, MAPK, and STAT-JAK represent the primary signaling pathways and transcription factors that regulate inflammatory responses. As an active innate immune reaction to microenvironment challenge, inflammation is an essential protective response to maintain homeostasis. However, with the failure to clear noxious inflammatory materials or apoptotic inflammatory cells, inflammation may become chronic and lead to pathological lesion or chronic inflammatory diseases, such as cancers, arthritis, and cardiovascular diseases [123].

#### 4.2.2. Long Non-Coding RNAs Promote the Inflammatory Response

Studies have demonstrated the crucial functions of lncRNAs in promoting gene transcription, protein modification, and chromatin accessibility during the inflammatory response triggered by PRR activation. The first evidence that lncRNA expression can be induced in innate immune cells came from the studies in TLR4-activated cells. The expression of *lincRNA-Cox2* (Table 2) is induced by more than 1000-fold in TLR4-stimulated CD11c^+^ bone-marrow-derived dendritic cells depending on the NF-κB pathway [124]. In addition, silencing of *lincRNA-Cox2* led to the attenuated expression of 713 genes following Pam3CSK4 (a TLR1/2 agonist) stimulation. Mechanistically, in LPS-stimulated macrophages, the assembly of *lincRNA-Cox2* into the switch/sucrose nonfermentable (SWI/SNF) complex is required for the incorporation of NF-κB subunits into the SWI/SNF complex, subsequently promoting histone H3 methylation and transactivation of late-primary inflammatory-response genes [125]. However, how *lincRNA-Cox2* “guides” the recruitment of SWI/SNF complex to NF-κB responsive loci is unclear, but probably occurs through RNA-DNA duplex formation between *lincRNA-Cox2* and the target gene loci. Despite its low basal expression level, the silencing of *lincRNA-Cox2* in resting macrophages increased the expression of 787 genes, most of which are involved in the inflammatory response, such as *Ccrl*, *Ccl5*, *Cx3cl1*, and IFN-stimulated genes (e.g., *Irf7*, *Oas1*, and *Isg15*). The proposed mechanism of *lincRNA-Cox2* for the transcriptional repression of the inflammatory genes is mediated by its interactions with hnRNP-A/B (encoded by Hnrnpab) and hnRNP-A2/B1 (encoded by Hnrnpa2b1), which inhibits inflammatory gene transcription in macrophages [11]. This research suggests that *lincRNA-Cox2* has a dual role in regulating immune responses, depending on the cell context.

*THRIL*, expressed in human tissues, is another lncRNA that participates in TNFα expression in response to stimulation with TLR2 ligand through forming an RNA–protein complex with hnRNP in THP1 macrophages [126]. The knockdown of *THRIL* decreased the expression of Pam-stimulated cytokines and chemokines including IL-8, TNFα, CCL1, CSF1, and CXCL10 among the more than 200 downregulated genes. Other proinflammatory lncRNAs, *MALAT1* [127] and *Sros1*, [128] promote the expression of proinflammatory mediators via the derepression of MyD88/NF-κB as an *miR-149* sponge in human lung injury inflammation and the activation of the STAT1 pathway by freeing *Stat1* mRNA from the RBP CAPRIN1, respectively.

#### 4.2.3. Long Non-Coding RNAs Inhibit the Inflammatory Response

Similar to the other non-coding RNAs, such as microRNAs that can be pro- or anti-inflammatory [141], lncRNAs have a dual effect on the regulation of innate immune responses. Transcriptome analysis demonstrates that *lincRNA-EPS* and *lnc13*, which are both localized in the nucleus, are two lncRNAs downregulated in macrophages after TLR activation, and repress the expression of inflammatory genes through association with chromatin at the regulatory sites of target genes, and binding to hnRNPD, respectively [10,135]. LPS-induced expression of chemokines (*Ccl4*, *Ccl5*, *Cxcl2* and *Cxcl10*), cytokines (*IL1α*, *IL6* and *IL15*), and antiviral ISGs (*Ifit1*, *Ifi204*, *Oas2* and *Rsad2/viperin*) are potently declined in *lincRNA-EPS* expressing BMDMs. These results were confirmed by an enhanced inflammation response in *lincRNA-EPS*-deficient mice in vivo [10]. Similarly, the expression of master regulators of the inflammatory response, including *TRAF2*, *MyD88*, *IL1RA*, and *STAT1*, were potently enhanced in patients with Celiac disease associated with low expression of *lnc13* in small intestine [135]. Another example of a lncRNA repressing inflammation is *Lethe*, a pseudogene lncRNA, primarily localized on the chromatin and highly induced by the proinflammatory cytokines IL-1β and TNF-α in mouse embryonic fibroblasts. *Lethe* functions as a negative feedback modulator of the NF-κB signaling pathway through interaction with NF-κB subunit RelA (p65), thus, preventing RelA from binding to the promoters of target genes, such as *IL6* and *IL8* [136]. In addition, *Lethe*-mediated blockage of RelA translocation into nucleus limits ROS production in macrophages, which may also contribute to the anti-inflammatory role of *Lethe*. The decreased *Lethe* expression and increased NADP oxidase gene expression observed in a mouse model of diabetic wound healing also support these findings [142]. *MALAT1*, another NF-κB repressor, restricts excessive inflammatory responses of LPS-activated macrophages by inhibiting NF-κB DNA binding activity [137]. Furthermore, as discussed above, *Mirt2* inhibits cytokine (e.g., IL-6, CXCL9) production through the inactivation of MAPK/NF-κB pathways in macrophages [99]. Collectively, these studies highlight the crucial role of lncRNAs in the negative regulation of inflammatory response, which may provide potential strategies for the treatment of inflammatory diseases.

### 4.3. Long Non-Coding RNAs Function in Antiviral Innate Immune Response 

In higher organisms, host antiviral innate immune response is triggered by the recognition of viral nucleic acids by PRRs, including TLR family members and the RLR family. After sensing viral invasion, a rapid induction of signaling cascades, such as type I and III IFN signaling [143], is initiated to coordinate innate immune cell behaviors with viral clearance. Although initial studies on the biological functions of lncRNAs in innate immunity primarily focused on host responses against bacteria, an increasing amount of research has demonstrated that thousands of lncRNAs are regulated by DNA or RNA virus infection. These lncRNAs may function to promote or inhibit viral replication and clearance by initiating or inactivating the induction of crucial viral sensors, IFN signaling, and the expression of direct viral clearance effectors. 

#### 4.3.1. Antiviral Signaling

Structural proteins of viral envelope and capsid are major PAMPs that are recognized by serval TLRs present in the cell membrane, such as TLR2 and TLR4, while viral RNA or DNA, when released into the cytoplasm of infected host cells, are recognized by endosomal TLRs: for example, TLR3 senses double-stranded RNA [144], TLR7/8 recognize degradation products of single-stranded RNA (ssRNA) [145], and TLR9 is specific to DNA with unmethylated CpG [146]. 

The RLRs are a family that detect cytosolic viral RNAs and are essential for the initiation of the innate immune response against RNA viruses. RLR sensors include three members: RIG-I, melanoma differentiation-associated gene 5 (MDA5) and laboratory of genetics and physiology 2 (LGP2), which are similarly organized and share a central DExD/H box helicase domain [147]. RIG-I and MDA5 have two N-terminal caspase activation and recruitment domains (CARDs) that are responsible for the interaction between activated RIG-I or MDA5 and the adaptor protein mitochondrial antiviral signaling (MAVS), which mediates the activation of NF-κB, IRF3, IRF7, and ATF2 in response to viral infection [143]. In addition, cyclic GMP-AMP synthase (cGAS) is another important sensor, which recognizes cytosolic double-stranded DNA and initiates the secretion of type I IFN and other inflammatory cytokines [148]. Moreover, the cytosolic viral DNA induces NLRP3 inflammasome complex formation through ROS, or AIM2 associated ASC adapter and pro-caspase-1, which converts pro-caspase-1 into its active form cleaving pro-IL-1β and pro-IL-18 into mature forms [149,150]. 

The common outcome of these signaling pathways is transcription of the inflammatory genes initiated by NF-κB or ATF2, and the transcription of important antiviral genes (such as type I IFN, *IFN-α*, and *IFN-β*) initiated by IRF3 or IRF7, responsible for viral clearance.

#### 4.3.2. Long Non-Coding RNAs Promote Antiviral Innate Immune Response 

Like the inflammatory signaling pathways, antiviral signaling is also strictly regulated by lncRNAs. Influenza A virus (IAV) is a common pathogen that causes respiratory tract infections and constitutes a major threat to human health. Several lncRNAs, such as *lncRNA-155*, RIG-I-dependent IAV-upregulated non-coding RNA (*RDUR*), and *NEAT1*, are upregulated in innate immune cells upon IAV infection. TLR3 and RIG-1 induced upregulation of *lncRNA-155*, both in vitro and in vivo (mouse model), promotes the production of *IFN-β* and several vital IFN-stimulated genes (ISGs), such as *Mx1*, *Isg15*, and *Oas3*, by inhibiting protein tyrosine phosphatase 1B (PTP1B) expression [129]. NF-κB-mediated increase in *RDUR* expression plays a similar role in promoting antiviral molecule expression (e.g., IFNs and ISGs) in vitro and in vivo; meanwhile, it inactivates NF-κB to prevent an excessive inflammatory response through a negative feedback mechanism [130]. *NEAT1* has been found to be associated with cytokine IL-8 expression in response to dsRNA-mediated TLR3 activation or viral infections, such as IAV and Herpes simplex virus type (HSV-1) infection. It initiates *IL-8* transcription by binding to and transferring the repressor splicing factor proline/glutamine-rich (SFPQ) from the *IL-8* promoter to the nuclear paraspeckle bodies [132]. *NEAT1* also promotes AIM2, NLRP3, or NLRP4 inflammasome assembly and maintains a mature caspase-1 for IL-1β production and pyroptosis [131].

Moreover, RIG-1 signaling is also enhanced by *Lnczc3h7a*, which forms one stable trimeric complex by binding to TRIM25 in macrophages upon the infection of RNA or DNA viruses and IFN-β stimulation. This complex acts as a scaffold to promote and stabilize the interaction between TRIM25 and the activated RIG-I, and then strengthens TRIM25-mediated K63-linked ubiquitination of RIG-I [133]. In epithelial A549 cells, researchers found that lncRNA *OASL-IT1* promotes the phosphorylation of p38 MAPK, IRF3, and NF-κB p65, leading to the expression of IFN-β and two classic ISGs (MX1 and IFITM1) in a positive feedback manner during Zika virus (ZIKV) infection [134]. 

#### 4.3.3. Long Non-Coding RNAs Inhibit Antiviral Innate Immune Response 

Most recently, an array of novel lncRNAs were found to regulate the virus-related innate immune response in a negative manner. Cytoplasmic *lncATV* is highly expressed in human monocytes, hepatoma cells, and erythroleukemia cells, and is upregulated upon type I/III IFN stimulation and infection of viruses, such as hepatitis C virus, Sendai virus, Newcastle disease virus, and ZIKV. *LncATV* potently inhibits RIG-I antiviral signaling and the IFN pathway, probably due to its association with RIG-I [138]. Similar to *lncATV*, lncRNA *lnc-Lsm3b*, which is upregulated upon virus infection, blocks RIG-1 activation through binding to the CARD and helicase domain of RIG-1, limiting RIG-I ubiquitination and phosphorylation, and reducing virus-induced *IFN-β* and *NF-κB* promoter activity. This was also confirmed in *lnc-Lsm3b*-deficient mice [139].

Different from *lncATV* and *lnc-Lsm3b*, *MALAT1* is downregulated in macrophages infected with viruses. A reduction in *MALAT1* expression is required for caspase-3-mediated TDP43 to TDP35 activation in nucleus, which prevents IRF3 from proteasomal degradation and promotes type I IFN production. Additionally, *MALAT1*-deficient mice show enhanced antiviral response after VSV or HSV-1 infection [151]. LncRNA *NRAV* inhibits the initial transcription of multiple critical ISGs, including *MxA* and *IFITM3*, by regulating the histone modification of these genes, and causes IAV replication and virulence in human cells and transgenic mice expressing human *NRAV*. It is demonstrated that the formation of the spatial structure of *NRAV* stem loops, except one small arm (nt 618-872), is necessary for *NRAV* biological function during virus infection [140]. 

## 5. Innate Immune Long Non-Coding RNAs in Non-Infectious Diseases

In addition to infectious diseases, inappropriately engaged or dysregulated inflammatory process may disturb tissue homeostasis and cause extensive autoimmune diseases, such as chronic auto-inflammatory diseases, atopic dermatitis, cardiovascular diseases, obesity, and type 2 diabetes [152]. Genome-wide association studies (GWAS) showed that more than 90% of disease-related SNPs occur in non-coding regions [153] and approximately 10% of SNPs associated with immune and autoimmune disorders are found in lncRNA loci [154]. Moreover, altered expression of lncRNAs has been found in several inflammatory diseases; thus, with future prospective studies, lncRNAs could represent a new therapeutic target for this type of disease.

### 5.1. Hematological Diseases

Prolonged inflammation caused by dysregulated innate immune cell survival leads to many human inflammatory and hematological diseases [155,156]; thus, the lifespan of innate immune cells must be strictly controlled. Hypereosinophilic syndrome (HES) is a kind of disorder characterized by eosinophilia associated with increased responsiveness to IL-5. A lncRNA *Morrbid*, elevated in some HES patients, plays a critical role in the development of HES by inhibiting the apoptosis of eosinophils [157]. *Morrbid* represses the transcription of its neighboring pro-apoptotic gene *Bcl2l11* by promoting the enrichment of PRC2 and subsequent deposition of repressive H3K27me3 at the bivalent promoter of *Bcl2l11* in short-lived myeloid cells, including neutrophils, eosinophils, and classical monocytes, in response to pro-survival cytokines, like IL-5. Moreover, impaired hematopoietic differentiation in leukemia may also be a result of dysregulated lncRNAs, for example, *HOTAIRM1* and *NEAT1*. As mentioned above, the lack of *HOTAIRM1* suppresses the activation of *HoxA1* and *HoxA4*, leading to granulocytic differentiation blockade in NB4 acute promyelocytic leukemia cell line [103]. Promyelocytic leukemia with retinoic acid receptor alpha (PML-RARα)-fusion-mediated *NEAT1* transcriptional repression might impair the myelopoiesis of acute promyelocytic leukemia cells [158]. Although these studies suggest a pathological function of lncRNAs in hematological diseases, most are based on in vitro experiments and further experimentation in vivo will be required. 

### 5.2. Rheumatoid Arthritis

Rheumatoid arthritis (RA) is one prevalent chronic inflammatory disorder, characterized by persistent synovitis in the joints [159]. Although the precise etiology of RA is still unclear, both genetic and environmental factors have been identified as important contributors to RA development. In recent years, several studies have revealed the functional role of lncRNAs in peripheral blood mononuclear cells (PBMCs) or fibroblast-like synoviocytes (FLSs) in RA pathology. *Hotari* is one of the firstly reported lncRNAs and notably expressed in the PBMCs and serum exosomes of RA patients [160]. The upregulated *Hotari* in RA exosomes may induce the migration of activated macrophages to the joints. Moreover, *Hotair* activates MMP-2 and MMP-13 in osteoclasts and synoviocytes, which may lead to proteinase-mediated dissolution of articular cartilage matrix and subchondral bone resorption during RA pathogenesis. In contrast, lncRNA *CASC2* is downregulated in the plasma of RA patients [161]. Through decreasing IL-17 expression, *CASC2* could potently promote the apoptosis of FLSs that contribute to RA development by producing cytokines and proteases [161]. However, due to the complicated pathogenic mechanism of autoimmune disease, there is extensive lncRNA regulatory potential that has yet to be discovered in RA development. 

### 5.3. Cardiovascular Diseases

Cardiovascular disease is a major cause of mortality and morbidity in patients with chronic inflammatory disorders, such as the RA described above. The cytokines and chemokines produced by innate immune cells during the chronic inflammatory response not only act as biomarkers but also directly contribute to the pathogenesis of cardiovascular diseases, where macrophages play a crucial pathological role [162]. Macrophage accumulation within vascular intima leads to persistent local inflammatory responses, causing atherosclerosis [163]. An array of lncRNAs have been reported in the tight regulation of macrophage phenotypes during the progression of atherosclerosis, such as lncRNA *RAPIA* [164], *MAARS* [165], *MIAT* [166], *PELATON* [167], *Mirt2* [99], by controlling the inflammatory process, lipid homeostasis, and cell cycle. LncRNAs also play an important role in maintaining the hemostasis of endothelial cells that are nonprofessional innate immune cells, during the development of cardiovascular diseases, such as lncRNA *SRA* [168], *NEXN-AS1/NEXN* [169], *lncRNA-CCL2* [170]. However, considering that cardiovascular disease is the leading cause of death worldwide, the current understanding of lncRNAs contributing to this pathogenic process is still insufficient. 

### 5.4. Intestinal Diseases

Inflammatory bowel disease (IBD) represents a group of intestinal disorders characterized by prolonged inflammation of the digestive tract and systemic release of the luminal microbiota due to an imbalance between the intestinal immune system and microbiota. A growing body of evidence has demonstrated that innate immune lncRNAs are involved in the pathogenesis of IBD, including ulcerative colitis and Crohn’s disease. In accordance with its proinflammatory role, genetic variants decreasing *Lnc-ROCKI* expression in human monocytes reduce the risk of IBD and other inflammatory diseases (such as atherosclerosis). [171]. However, most research into lncRNA functions in IBD focuses on nonprofessional innate immune cells, such as intestinal epithelial cells (IECs). A lncRNA *HIF1A-AS2* is upregulated in mice with ulcerative colitis induced by Flagellin and inhibits cytokine expression in IEC-like cells as well as alleviating colonic inflammation in vivo [172]. The suppression of *ANRIL* alleviated LPS-induced injury in fetal human cells and inhibited the development of ulcerative colitis through the TLR4/MyD88/NF-κB pathway by negatively regulating *miR-323b-5p* [173]. 

Celiac disease (CeD), a chronic and innate immune-mediated intestinal disorder, is closely associated with non-coding regions of human genome [174,175]. Studies have revealed that *lnc13* levels are decreased in small intestine of CeD patients, repressing the expression of inflammatory genes (e.g., *Stat1*, *Stat3*, *Traf2*, *Myd88*, *Ccl12*, *Il1ra*) in macrophages, indicating a potential downregulation role for *lnc13* in the pathogenesis of CeD [135]. Other lncRNAs, such as *Neat1* and *TUG1* also participate in DeD disease by association with STAT3 [176].

### 5.5. Diabetes Mellitus

Diabetes mellitus (DM) is a metabolic disorder characterized by chronic low-grade inflammation in the pancreatic islets and impaired insulin secretory capacity. Over the last decade, human transcriptome analyses have shown that lncRNAs are dynamically regulated and abnormally expressed in patients with DM, such as *MALAT1*, *uc.48+*, *E330013P06*, *Hotair*, *Miat*, and *GAS5*, and might be potential diagnostic biomarkers for DM [177,178,179,180]. *MALAT1* was found to improve DM-induced retinal endothelial cell dysfunction and microvascular abnormalities by activating the p38/MAPK signaling pathway [177]. A lncRNA *uc.48+* are responsible for the development of type 2 diabetes (T2DM) by inducing P2X_7_P-mediated immune, ERK1/2-mediated proinflammatory response, and ROS formation in the RAW264.7 macrophage [178]. Moreover, *E330013P06* contributes to the increased susceptibility of T2DM through the enhancement of the inflammatory response and macrophage-derived foam cell formation [179]. However, the functional role of lncRNAs in innate immune cells during DM development is still being established.

## 6. Concluding Remarks and Future Perspectives

Tremendous progress made in recent years has provided clear evidence that lncRNAs play an important role in the regulation of innate immunity. Although we have focused on exploring the functional roles and mechanisms of these RNAs, a large void in our understanding of how these lncRNAs function at the molecular level in the context of innate immune response remains. The innate immune response initiates with the recognition of pathogens through PRRs by innate immune cells accompanied by the development of inflammatory response. A large amount of inflammation-related signaling pathways and molecules, including classical TLRs, NF-κB, cytokines, and chemokines, are involved in this progress. Studies on lncRNA biology and functions greatly expanded our knowledge of how genes associated with inflammation and innate immune response are regulated. Various lncRNAs participate in the development and differentiation of innate immune cells, key molecule transcription, signaling transduction and disease development through versatile mechanisms, in either a positive or negative pattern. Nevertheless, given its complexity, what determines the net effect that the incorporated network between regulatory lncRNAs and inflammatory signaling pathways has on the pathophysiological fate of the immune system remains to be fully understood.

In addition, lncRNAs regulate transcriptional programs through diverse mechanisms including interactions with chromatin, DNA, RNA, and proteins either independent of or dependent on specific sequences. Less sequence conservation is not necessarily equivalent to fewer biological functions. However, we still have a poor understanding of how selective pressures act on lncRNAs at the sequence and structural levels, raising questions about what determines the evolution and function of lncRNAs. For instance, what are the key motifs or signatures for lncRNAs that have tissue- or cell-specific functions depending on their sequences? What is the role of alternative splicing for those lncRNAs that function through their transcript and processing independent of sequences? Do lncRNAs that share similar functions have similar and specific features? Additionally, a small number of lncRNAs are found to encode small peptides, which has not been investigated in detail. Finally, since the number of described lncRNAs is rapidly increasing due to the wide-range application of high-throughput sequencing technologies, an illustration of the exact molecular mechanisms underlying the biological functions of lncRNAs will be the greatest challenge in lncRNA studies.

## Figures and Tables

**Table 2:** Long non-coding RNAs act as modulators of inflammatory responses in innate immunity.

Model	LncRNA	Functional Consequences	Mechanism	Reference
Positive pattern	LincRNA-Cox2	Transactivates inflammatory genes	Incorporates NF-κB into the SWI/SNF complex	[125]
	THRIL	Promotes TLR2-mediated cytokines and chemokines expression	Forms an RNA-protein complex with hnRNP	[126]
	MALAT1	Promotes IL-1β, IL-6 and TNF-α expression	Sponges miR-149	[127]
	LncRNA Sros1	Promotes IFN-γ-STAT1-mediated innate immunity	Frees STAT1 mRNA from the RBP CAPRIN1	[128]
	LncRNA-155	Promotes IFN-β and ISGs production	Inhibits PTP1B production	[129]
	RDUR	Upregulates IFNs and ISGs expression, alleviates inflammation	Inactivates NF-κB	[130]
	NEAT1	Promotes inflammasomes assembly, initiates antiviral gene IL-8 transcription	Transfers the SFPQ from IL-8 promoter, maintains caspase-1 maturation	[131,132]
	Lnczc3h7a	Activates TRIM25-mediated RIG-I antiviral response	Forms trimeric complex with RIG-I and TRIM25	[133]
	OASL-IT1	Triggers IFN-β and ISGs expression, inhibits ZIKV infection	Activates p38 MAPK, IRF3, and NF-κB	[134]
Negative pattern	LincRNA-Cox2	Represses inflammatory response	Interaction with hnRNP-A/B and hnRNP-A2/B1	[11]
	LincRNA-EPS	Represses inflammatory genes expression	Interaction with chromatin, hnRNPD or histone	[10]
	Lnc13	Decreases inflammatory regulators expression	Binds to hnRNPD p42 and Hdac1 on chromatin	[135]
	Lethe	Prevents proinflammatory cytokines production	Prevents RelA-mediated transcription	[136]
	MALAT1	Prevents proinflammatory cytokines and IFN-I production	Binding to NF-κB, prevents IRF3 degradation	[137]
	Mirt2	Inhibits cytokine (e.g., IL-6, CXCL9) production	Inactivates MAPK/NF-κB pathways	[99]
	LncATV	Inhibits IFNs and ISGs production	Induces a mono-allelic mutation in the CARD of RIG-I	[138]
	Lnc-Lsm3b	Terminates type I IFNs production	Limits RIG-I ubiquitination and phosphorylation	[139]
	NRAV	Inhibits transcription of ISGs	Regulation on histone modification	[140]

## Data Availability

Not applicable.
